# Grey Correlation Analysis of Drying Characteristics and Quality of *Hypsizygus marmoreus* (Crab-Flavoured Mushroom) By-Products

**DOI:** 10.3390/molecules28217394

**Published:** 2023-11-02

**Authors:** Pufu Lai, Zheng Xiao, Yibin Li, Baosha Tang, Li Wu, Minjie Weng, Junzheng Sun, Junchen Chen

**Affiliations:** 1 Institute of Food Science and Technology, Fujian Academy of Agricultural Sciences, Fuzhou 350013, China; ethyxwat@163.com (Z.X.); lyb9951@163.com (Y.L.); tbsty@126.com (B.T.); xxj1963@163.com (L.W.); fjsxwmj@gmail.com (M.W.); sunjzll@163.com (J.S.); junchencc@sina.com (J.C.); 2 National R&D Center for Edible Fungi Processing, Fuzhou 350003, China; 3 Key Laboratory of Subtropical Characteristic Fruits, Vegetables and Edible Fungi Processing (Co-Construction by Ministry and Province), Ministry of Agriculture and Rural Affairs, Fuzhou 350003, China

**Keywords:** *Hypsizygus marmoreus*, by-products, drying methods, quality, grey correlation analysis

## Abstract

The physical properties and nutritional quality of *H. marmoreus* by-products (HMB) dried by different methods were comprehensively evaluated by a rigorous statistical method of grey correlation analysis. The results indicated that different drying methods had significant impacts on the characteristics of HMB. Heat pump drying (HPD) was conducive to the preservation of protein and reducing sugar, and hot air drying (HAD) maintained a high content of total flavonoids. The highest fat, polysaccharide, and total phenolic contents were obtained by heated vacuum freeze-drying (H-VFD) treatment. The unheated vacuum freeze-drying (UH-VFD) treatment achieved bright colour, lacunose texture profile, and looser organization structure. The grey correlation analysis showed that UH-VFD and H-VFD had higher-weighted correlation degrees than HPD and HAD. HMB had many higher nutritional components than commodity specifications, especially protein, fat, polyphenols, and amino acids, and had potential applications in the food industry as functional foods and nutraceutical agents.

## 1. Introduction

Mushrooms with unique flavours and rich nutrients are important agricultural products all around the world. The global mushroom market is increasing yearly and is projected to grow from 15.25 million tonnes in 2021 to 24.05 million tonnes in 2028 at a compound annual growth rate of 6.74% [1]. Unfortunately, a large number of by-products appear in the current edible mushroom industry, such as damaged, defective, or small fruiting bodies. The by-products of edible fungi contain rich polysaccharides and protein nutrients for the human body, which have excellent utilization value and development potential [2]. For instance, the by-products of *Pleurotus eryngii* have a similar crude polysaccharide content compared with the fruiting body [3]. The nutritional index of *Lentinus edodes* by-products is equivalent to that of the fruiting body, and the flavouring substances’ content is much higher [4]. *Hypsizygus marmoreus*, also known as crab-flavoured mushroom, is rich in protein, flavonoids, polysaccharides, phenols, and trace elements and exhibits the activities of anti-oxidation, anti-inflammatory, anti-tumour, reducing blood lipid, and immune regulation [5]. However, 5~10% of by-products can be produced during its harvesting and processing, especially small fruiting bodies that do not meet the commodity standards. At present, most of them are directly discarded as waste.

The small fruiting bodies of edible mushrooms often contain more nutrients than commercial commodities, although they are not popular for fresh eating. They can be used in the food industry after drying. Drying is a common preservation technology employed in the processing and preservation of many predominant agricultural products such as meat, fruits, and vegetables [6,7,8]. The widely used process can reduce the water content and water activity (AW) to a certain extent, thus effectively solving the short shelf life problem and reducing storage and transportation costs [9,10]. In addition, the drying method directly affects the main nutrition components, active substances, appearance, and physical characteristics. Therefore, selecting an appropriate drying method to retain more nutrients, active substances, and excellent physical properties is particularly important.

As a kind of macrofungi, the fruiting body of edible fungi consists of different parts with different nutrient contents. Drying methods have different effects on different parts of food [11]. Therefore, an in-depth study of the nutritional composition and physical characteristics of different parts of edible fungi after different drying methods is necessary to develop various products targeting to increase their commercial value.

The previous literature has usually evaluated different drying methods through several parameters but lacked rigorous statistical methods for comprehensive evaluation. Compared with the traditional analysis methods such as variance analysis or regression analysis, which only take a single indicator as the measurement standard, the grey correlation analysis can comprehensively and genuinely reflect the changes of comprehensive factors by comparing multiple trait indicators as a whole, and its comprehensive evaluation results are more scientific and accurate [12]. In recent years, grey relational analysis has been widely used in the comprehensive evaluation of various agricultural products [13,14].

**Figure 1:**
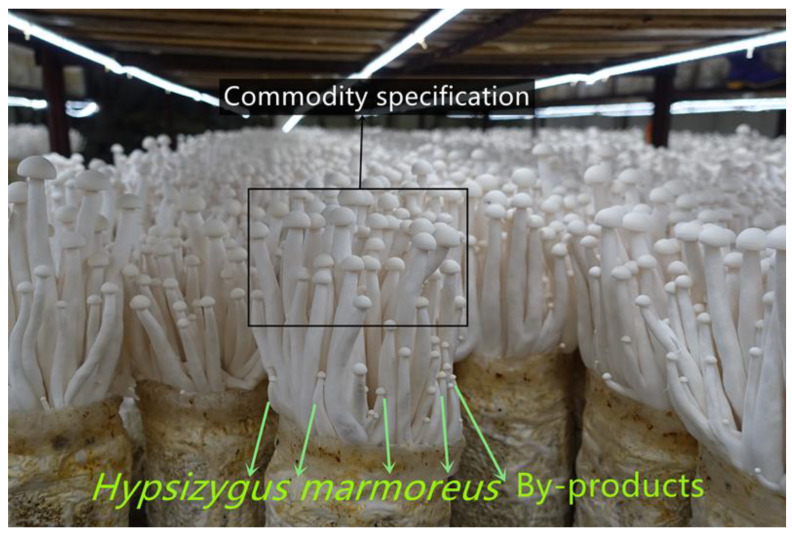
*Hypsizygus marmoreus* commodity specification and by-products (small fruiting bodies).

In this study, unheated vacuum freeze drying (UH-VFD), heated vacuum freeze drying (H-VFD), heat pump drying (HPD), and hot air drying (HAD) were performed on *H. marmoreus* by-products (HMB; Figure 1), respectively. Their effects on the nutrient components, active substances, colour difference, texture, and microstructure of HMB were compared. The physical properties and nutrient components were analysed comprehensively by using the grey correlation method. The results provide a theoretical basis for the further processing and utilization of HMB.

## 2. Results and Discussion

### 2.1. Effects of Different Drying Methods on Nutritional Components of HMB

**Figure 2:**
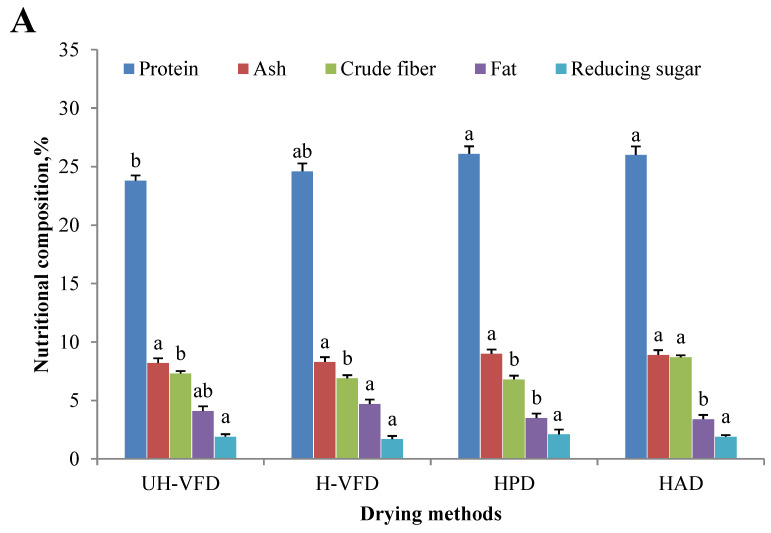

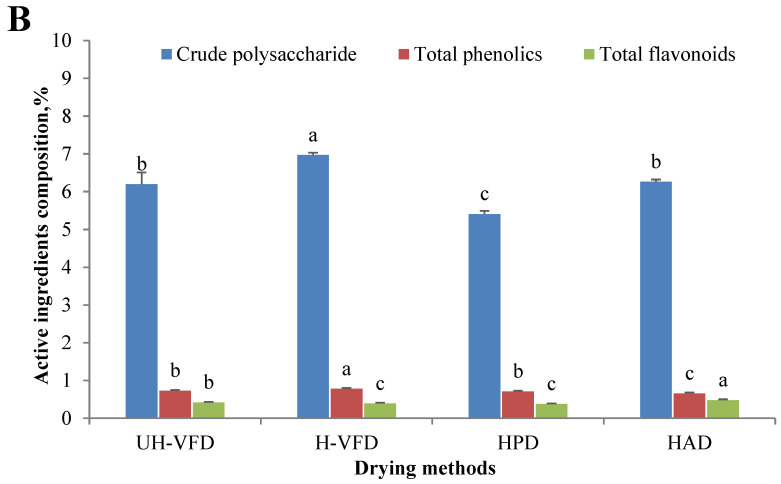
Composition of *Hypsizygus marmoreus* by-products prepared by different drying methods. (**A**) Main chemical properties; (**B**) bioactive properties. Different letters in the same indicators indicate significant differences (*p* < 0.05), as shown below.

Nutrient composition, including protein, ash, fat, reducing sugar, etc., is an essential indicator for measuring the value of mushroom products. Due to the change in cell membrane osmotic pressure and the action of related enzymes, the protein, starch, carbohydrate, and other nutrient components of fruit and vegetable cells are decomposed into small molecular substances during the drying process, which causes changes in the content of related components [15]. The drying method has a particular influence on dried products’ nutrition and active ingredients. The changes in the main nutritional features of HMB under different drying treatments are shown in Figure 2A. The protein content of the UH-VFD (23.8%) and H-VFD (24.6%) was significantly lower than that of the HPD (26.1%) and HAD (26.0%). This may be due to the denaturation of hydrogen bonds and polar groups of water molecules on the protein surface due to environmental changes in the VFD process without protective agents [16]. The crude fibre content of HAD group was significantly higher than that of other drying methods. This might be due to the long-term thermal action that promotes the conversion of soluble fibres in HMB into insoluble fibres. The effects of different drying methods on ash, fat, and reducing sugar were insignificant.

### 2.2. Effects of Different Drying Methods on the Active Ingredients of HMB

According to Figure 2B, the effects of different drying treatments on the contents of total flavonoids, total polyphenols, and crude polysaccharides were significantly different. The total polyphenols and crude polysaccharides of VFD were considerably higher than those of HPD and HAD. This might be due to the high-temperature process of HPD and HAD, which caused HMB tissue cells to break down, releasing hydrolase and oxidase. Then, the oxidation of polyphenols, polysaccharides, and other substances occurred. At the same time, during the VFD process, the isolation of oxygen slowed down the occurrence of biochemical reactions of polyphenols and sugars and, finally, reduced their consumption. The H-VFD group’s total polyphenols and crude polysaccharides were 0.78% and 6.97%, respectively, the highest among the four drying methods. In the VFD process with auxiliary heating, the heating enabled the nonenzymatic conversion of phenolic molecules into polyphenols, increasing the content of polyphenols and carbohydrates. The content of total flavonoids of the HAD group was significantly higher than those of the other three groups. Flavonoids usually coexist with carbohydrates, proteins, etc. Heating promotes cell fragmentation and covalent bond breakage, which is conducive to the dissolution of flavonoids [17].

### 2.3. Effects of Different Drying Methods on the Amino Acid Composition of HMB

Amino acids, the basic components of protein, are essential nutrient elements for the human body. It can be seen from Table 1 that the total amino acid content, essential amino acid content, and nonessential amino acid content of VFD groups were significantly lower than that of the HPD and HAD groups (*p* < 0.05). Still, the difference between HPD and HAD was not significant (*p* > 0.05). This might be due to the high drying temperature contributing to the release of amino acids [18]. HMB processed by four drying methods contained 17 kinds of amino acids (tryptophan not measured) and 7 kinds of amino acids necessary for the human body. The primary amino acids were methionine, glutamic acid, and aspartic acid. The composition and content of umami amino acids determine the taste of the sample [19]. HMB contained seven kinds of umami amino acids: glutamic acid, aspartic acid, arginine, histidine, proline, glycine, and alanine. Their high content forms the seafood flavour of HMB. The proportion of essential amino acids to total amino acids (EAA/TAA) was 0.43, 0.43, 0.41, and 0.43, respectively. The ratio of total essential amino acids to total nonessential amino acids (EAA/NEAA) was more than 0.7, which conformed to the reference protein model proposed by FAO [20]. These results indicated that HMB was a good source of high-quality protein.

### 2.4. Effects of Different Drying Methods on Color Indices of HMB

**Figure 3:**
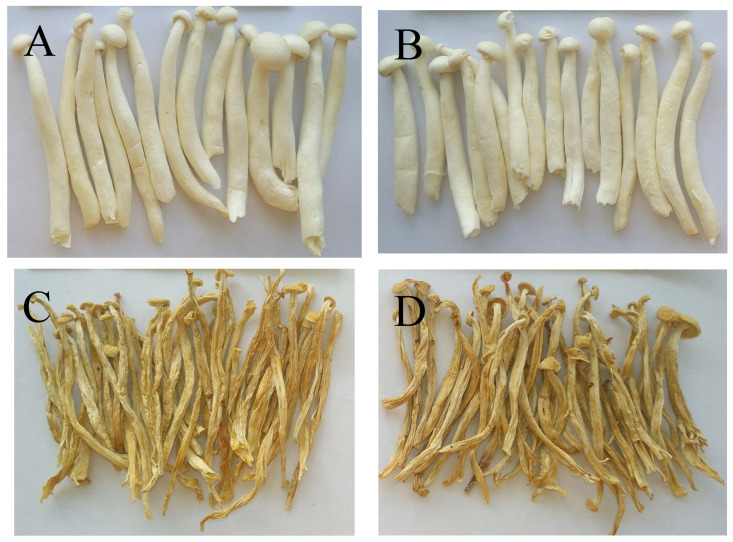
Extrinsic shapes of *Hypsizygus marmoreus* by-products prepared by different drying methods: (**A**) unheated vacuum freeze drying (UH-VFD), (**B**) heated vacuum freeze drying (H-VFD), (**C**) heat pump drying (HPD), and (**D**) hot air drying (HAD).

**Figure 4:**
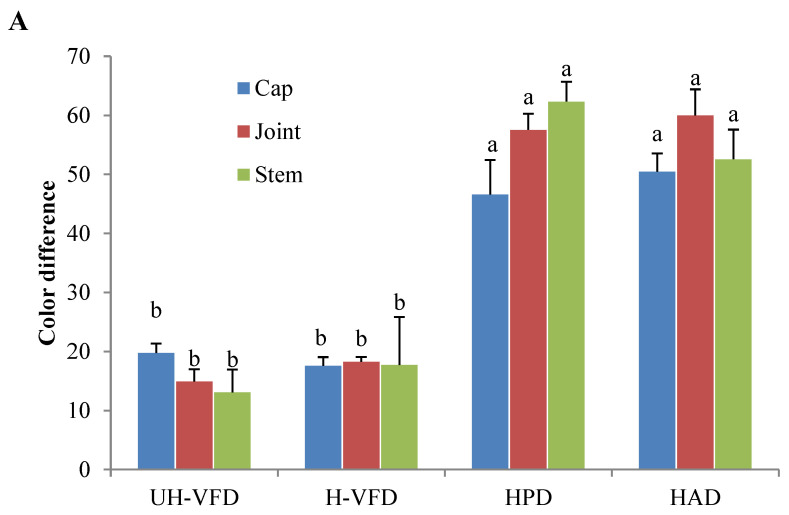

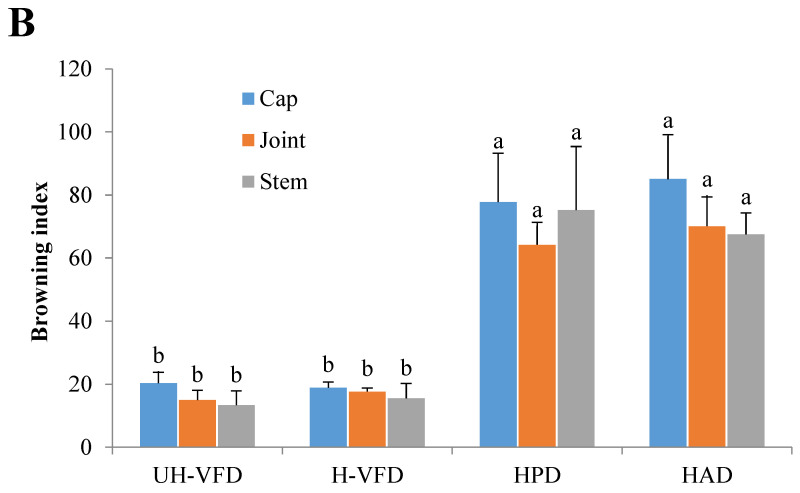
Colour indices of *Hypsizygus marmoreus* by-products prepared by different drying methods. (**A**) Colour difference; (**B**) browning index. Different letters in the same indicators indicate significant differences (*p* < 0.05).

Colour is an important quality attribute affecting consumer acceptance and market value. It can be seen from Figure 3 and Figure 4 that ΔE and BI of the UH-VFD group and H-VFD group had no significant difference, and the same applied to the HPD and HAD groups, but their ΔE and BI were significantly higher than those of the VFD groups. The colour of VFD groups was closer to that of fresh samples, indicating that VFD was a suitable drying method to maintain the original colour of HMB. The HMB dried by the four methods had browning to some extent. The colour of HMB treated by VFD was closer to that of fresh HMB, indicating that VFD can better maintain the original colour. This might be because the sample did not easily undergo the Maillard reaction due to enzyme passivation under vacuum conditions, which can better maintain the original colour of the sample [21].

### 2.5. Effects of Different Drying Methods on Texture Properties of HMB

**Figure 5:**
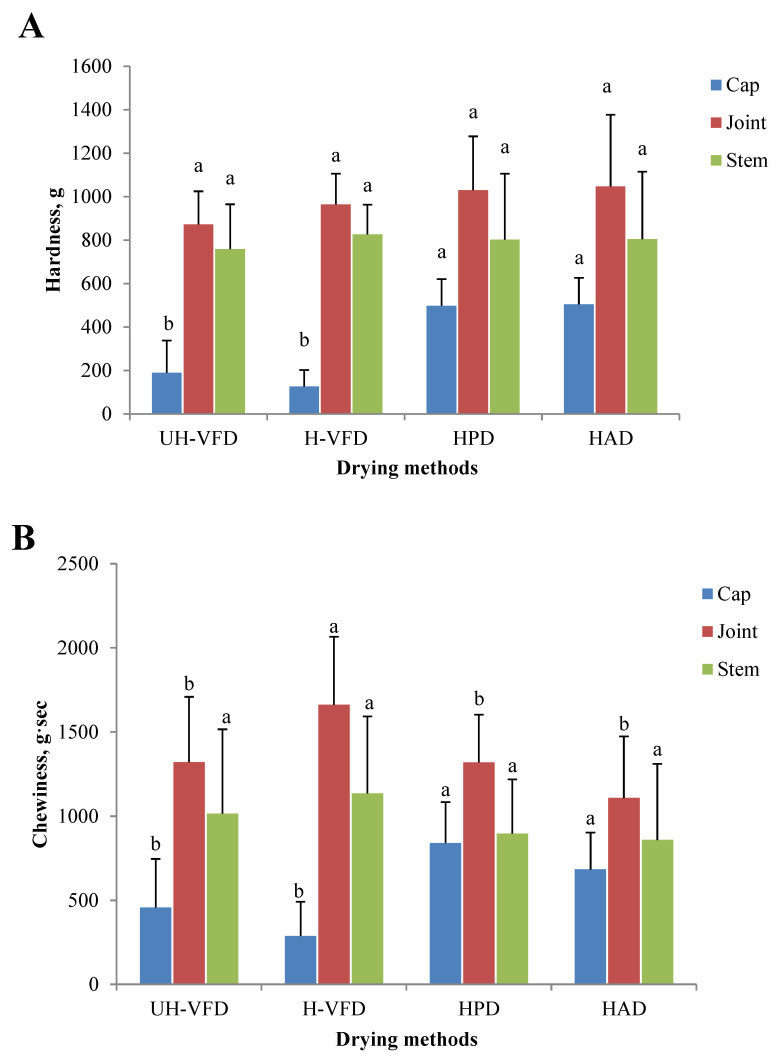
Texture properties of *Hypsizygus marmoreus* by-products prepared by different drying methods. (**A**) Hardness; (**B**) chewiness. Different letters in the same indicators indicate significant differences (*p* < 0.05).

The texture properties of HMB treated by different drying methods are given in Figure 5. The hardness and chewiness of HMB’s cap, joint, and stem samples were different. The joint had the highest hardness and chewiness, followed by the stem and the cap. It can be seen from Figure 5A that the hardness of the joint and stem had no significant difference, but the hardness of the cap after VFD treatment was significantly lower than that of HPD and HAD treatment. The moisture transfer rate is the main influencing factor of material hardness during drying. Moisture is rapidly removed in the drying process, forming a network structure and causing tissue fibre shrinkage. HAD makes the evaporation rate of water on the material surface greater than the internal section, and a layer of dry, hard film is formed on the surface, making the sample’s hardness greater. Due to the direct sublimation of ice crystals between cells of VFD products, the organizational structure is relatively complete, with a low shrinkage rate and large pore size, resulting in low hardness [22]. A similar change rule in the chewiness analysis is shown in Figure 5B. At the same time, the chewiness of the stem of the H-VFD group was significantly higher than that of other treatments.

### 2.6. Effects of Different Drying Methods on the Microstructure of HMB

**Figure 6:**
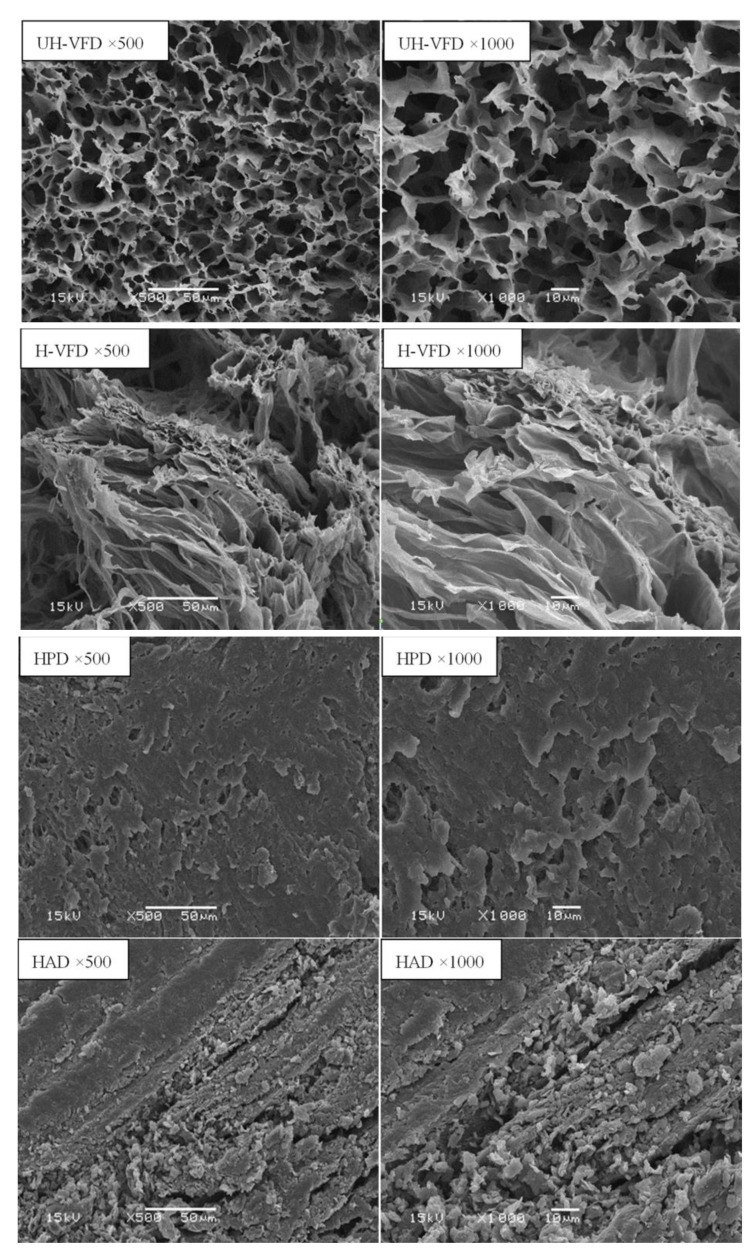
Micro-structure of *Hypsizygus marmoreus* by-products prepared with different drying methods.

Figure 6 shows the structural scanning diagram of HMB under different drying methods with the cross-section magnified 500 times and 1000 times. The cross-section of the UH-VFD group appeared to have large gaps, loose tissue, and uniform, porous honeycomb structures. The cross-section structure of the sample treated by H-VFD was also loose and porous, but there was a slight collapse phenomenon locally. The cross-section of the HPD group had loose structures and small gaps. HMB treated by HAD had a compact cross-section structure, severe tissue shrinkage and deformation, and obvious small slags. In the drying process, with the migration of water, materials produce capillary shrinkage, cell breakage, and other phenomena, leading to the tissue structure’s collapse. Krokida et al. studied the difference in bulk density and porosity of bananas, carrots, potatoes, and apples under different drying methods and found that VFD treatment had the lowest bulk density and the highest porosity [23]. VFD maintains a good network structure, which is consistent with the results of this study on the influence of different drying methods on the microstructure of HMB.

Relevant studies show that the drying method and process parameters determine the shrinkage range and internal damage degree of tissues and cells caused by drying and dehydration [24]. During drying, the quality of samples will be affected by temperature, vacuum degree, and other factors. Under high-temperature conditions, the chemical or physical changes of sample components will occur, and the colour and texture will also change accordingly. In contrast, under low-temperature conditions, the chemical reaction rates will decrease. The vacuum condition can inhibit the related oxidation reaction due to the absence of oxygen.

### 2.7. Comprehensive Evaluation of the Effects of Different Drying Methods on the Quality of HMB

#### 2.7.1. Analysis of the Weight of Each Indicator

To avoid the non-objectivity of equal weight distribution and eliminate the dimensional difference of each index, different weights of 13 evaluation indexes of dried HMB were determined using the variation coefficient method. The weighted analysis of each indicator is shown in Table 2.

#### 2.7.2. Dimensionless Processing

According to the index data, the reference series R_0_ was constructed. To avoid the index difference of each dimension, the original data were uniformly processed according to the grey system theory:

R_1_(1)/R_0_(1), R_2_(2)/R_0_(2), ……, R_n_(i)/R_0_(i), ……, i *=* 1, 2, 3, 4……13, n = 1, 2, 3, 4.

The results are shown in Table 3.

#### 2.7.3. Correlation Analysis

Based on the data in Table 3, the absolute difference between the reference sequence and the corresponding points (comparison sequence) was calculated by the following Equation (1):

Δ_n_(i) = |R_0_(i) − R_n_(i)| (1)

where Δ_n_(i) is the absolute difference; R_0_(i) is the reference sequence; R_n_(i) is the comparison sequence.

The results are shown in Table 4.

Correlation coefficient ξ_n_ (i) and relational degree Y were calculated by the following Equations (2) and (3), respectively:

ξ_n_ (i) = (Δ_n_ (i) _min_ + 0.5Δ_n_ (i) _max_)/(Δ_n_ (i) + 0.5Δ_n_ (i) _max_) (2)

where ξ_n_ (i) is the correlation coefficient; 0.5 is the distinguishing coefficient; the maximum difference Δ_n_ (i) _max_ = 3.580, and the minimum difference Δ_n_ (i) _min_ = 0.000.

(3) $$Y=\sum_{i=1}^{n}\xi_{n}(K)\times Q_{N}(K).$$

where Y is the relational degree; ξ_n_ (i) is the correlation coefficient; Q_N_ (K) is the weight of each indicator.

According to the principle of grey correlation analysis, the higher the correlation degree, the closer the comparison sequence to the reference sequence. Previous studies generally believe that VFD is a preferred drying technology in the food industry due to the higher quality of the final products than conventional drying methods [25]. It can be seen from Table 5 that the weighted correlation degree of the four drying methods in descending order is as follows: UH-VFD > H-VFD > HPD = HAD. The difference in weighted correlation between UH-VFD and H-VFD was not significant; both were much better than HPD and HAD.

### 2.8. Comparison of the Components between HMB and H. marmoreus of Commodity Specifications

Table 6 compares the components between HMB and *H. marmoreus* of commodity specifications (HMCS). The content of protein, fat, ash, crude fibre, total polyphenols, crude polysaccharides, and total amino acids in HMB is higher than that in HMCS. Protein and fat are essential nutritional components of edible mushrooms. Compared with HMCS, the contents of HMB protein and fat were 39.27% and 67.86% higher, respectively, while the contents of reducing sugar and crude fibre were similar. Regarding functional components, the total phenolic content of HMB was 1.59 times that of HMCS. The reason may be that the fruiting body of small mushrooms has higher metabolic activity. Phenolic compounds are minor metabolites usually present in mushroom species with potential advantages for human health, such as antioxidant, anti-inflammatory, antitumor, antihyperglycaemic, anti-osteoporotic, anti-tyrosinase, and antimicrobial activities [26]. The HMB, rich in nutritional and bioactive components, have many potential applications in the food industry as functional foods and nutraceutical agents for providing better health conditions.

The component data of *Hypsizygus marmoreus* of commodity specifications (HMCS) was obtained from Lai et al. [27]. Both products were dried by H-VFD.

## 3. Materials and Methods

### 3.1. Materials

The HMB were provided by Fujian Tongxing Mushroom Industry Co., Ltd. (Sanming, Fujian, China). They were small fruiting bodies (moisture content 88.6%, cap diameter less than 1.5 cm, and stalk length less than 4 cm) that failed to meet the commodity specifications after 110–120 days of normal growth.

### 3.2. Methods

#### 3.2.1. Sample Preparation

The fresh HMB were washed to remove surface impurities. Then, they were weighed in four portions of 3000 g each, and the four drying methods were performed, respectively.

UH-VFD: The fresh HMB were evenly spread on the tray in a refrigerator for pre-freezing at −40 °C for 12 h and then placed in a vacuum freeze dryer (SCIENTZ-30ND, Xinzhi Freeze-drying Equipment Co., Ltd., Ningbo, China) for drying. The final moisture content was 4.99%.

H-VFD: Auxiliary heating was used during VFD, and the shelf temperature was set as 60 °C. The final moisture content was 4.92%.

HPD: A heat pump dryer (ZWH-KFX-BT12, Xuefeng Refrigeration Equipment Co., Ltd., Ningde, China) was used, and the temperature was set at 50 °C. The final moisture content of the samples was 5.33%.

HAD: A constant temperature blast-drying oven (101A, Shanghai Experimental Instrument Factory Co., Ltd., Shanghai, China) was used, and the temperature was set at 60 °C. The final moisture content of the samples was 5.41%.

#### 3.2.2. Index Determination

(1) Moisture

Moisture content was determined by removing moisture at 105 °C and then calculated by weight loss as a percentage of the initial weight.

(2) Protein

According to the National Food Safety Standard of China (GB 5009.5-2016) [28], protein content was determined by the Kjeldahl method. Samples weighing 2 g were mixed with 0.4 g of copper sulphate, 6 g of potassium sulphate, and 20 mL of sulfuric acid in digestion tubes. Then, the tubes were put into the furnace for digestion. When the temperature reached 420 °C, the digestion lasted for one hour. After the sample was taken out and cooled, 50 mL of water was added, and the samples were measured on an automatic Kjeldahl nitrogen analyser (GK-600, GreenKerry, Heze, China).

(3) Amino acid

Amino acid content was determined according to the National Food Safety Standard of China (GB 5009.124-2016) [29]. Samples weighing 0.2 g were weighed into hydrolysis tubes and mixed with 10 mL of 6 mol/L hydrochloric acid solution. After being frozen for 5 min, the hydrolysis tubes were vacuumed, filled with nitrogen three times, sealed, and placed in an electric blast constant temperature box at 110 ± 1 °C for 22 h. After cooling, the hydrolysate was filtered and diluted to 50 mL. Then, 1.0 mL of filtrate was accurately transferred into a 15 mL test tube and dried under reduced pressure at 40–50 °C. The residue was dissolved in 2 mL of water, dried under reduced pressure, and evaporated to dryness. The residue was then dissolved in 2 mL sodium citrate buffer solution (pH 2). After filtration through the 0.22 μm membrane, the solution was transferred to the instrument injection bottle. The mixed amino acid standard working solution and sample solution were injected into the amino acid analyser (L-3000, Huameichen, Wuxi, China) at the same volume. The concentration of amino acids in the sample solution was calculated using the external standard method.

(4) Reducing sugar

Reducing sugar content was measured using the direct titration method according to the National Food Safety Standard of China (GB 5009.7-2016) [30]. Samples weighing 5 g were added to a 250 mL volumetric flask and dissolved in 50 mL water. Then, 5 mL of zinc acetate solution and 5 mL of potassium ferrocyanide solution were slowly added. Water was added to the scale of the volumetric flasks and mixed evenly with the samples. The mixture was left for 30 min. Then, the sample solution was filtered using dry filter paper. The initial filtrate was discarded, while the subsequent filtrate was retained. Next, 5.0 mL of alkaline copper tartrate solution A and B were added to a 150 mL conical flask, followed by 10 mL of water and 2–4 glass beads. The sample solution was added to the conical flask from the burette. The solution in the conical flask was heated to boiling within 2 min, kept boiling, and continued to titration at a rate of 1 drop/2 s until the blue colour faded as the endpoint.

The sample’s reducing sugar content is calculated according to Equation (4).

(4) $$X=\frac{m_{1}}{m\times F\times V/250\times 1000}\times 100\%$$

In the equation, X is the content of reducing sugar in the sample (%), $m_{1}$ is the mass of alkaline copper tartrate solution equivalent to reducing sugar (mg), m is the sample mass (g), F is the coefficient (0.8), V is the volume of consumed sample solution (mL), and 250 is the constant volume (mL).

(5) Fat

Fat content was measured using the Soxhlet extractor method according to the National Food Safety Standard of China (GB 5009.6-2016) [31]. Samples weighing 5 g were transferred into the filter paper cylinders and placed in the extraction cylinder of the Soxhlet extractors. The samples were continuously refluxed and extracted with petroleum ether for six hours. Then, the petroleum ether was recycled. The receiving bottles were evaporated to dryness on a water bath, then dried at 100 ± 5 °C for one hour, cooled in dryers, and weighed. The fat content in the sample was calculated according to Equation (5):

(5) $$X=\frac{m_{1}-m_{0}}{m_{2}}\times 100\%$$

In the equation, X is the content of fat in the sample (%),$m_{1}$ is the content of the receiving bottle and fat (g), $m_{0}$ is the mass of the receiving bottle (g), $m_{2}$ is the mass of the sample (g), and 100 is the conversion factor.

(6) Ash

Ash content was determined according to the National Food Safety Standard of China (GB 5009.4-2016) [32]. The samples were first heated on an electric heating plate to fully carbonise until smokeless, then placed in a high-temperature furnace and burned at 550 ± 25 °C for four hours. Then, the samples were cooled and weighed. The ash content in the sample was calculated according to Equation (6):

(6) $$X_{2}=\frac{m_{1}-m_{2}}{(m_{3}-m_{2})\times \omega }\times 100\%$$

In the formula, X_2_ is the ash content in the sample (%), $m_{1}$ is the mass of the crucible and ash (g), $m_{2}$ is the mass of the crucible (g), $m_{3}$ is the mass of the crucible and sample (g), and ω is the dry matter content (%) of the sample.

(7) Crude fibre

Crude fibre content was measured according to the determination of crude fibre in plant food (GB/T 5009.10-2003) [33]. Samples weighing 5 g were first digested with 200 mL of boiled sulfuric acid (1.25%) for 30 min. Then, the remaining residue was filtered with linen cloth and washed with boiling water until the washing solution was not acidic. The remaining residue on the linen cloth was washed into the original conical flask with 200 mL of boiled 1.25% potassium hydroxide solution. After slightly boiling for 30 min, the solution was immediately filtered, and the remaining residue was washed three times with boiling water, followed by ethanol and ether in sequence. The remaining residue was weighed after drying in an oven at 105 °C. The result is calculated according to Equation (7).

(7) $$X=\frac{G}{m}\times 100\%$$

In the equation, X is the content of coarse fibres in the sample (100%), G is the mass of the residue (g), and m is the mass of the sample (g).

(8) Crude polysaccharides

The content of crude polysaccharides was measured using the phenol-sulfuric acid method [34]. Samples weighing 0.5 g were thoroughly mixed with 5 mL of water, and then, 20 mL of anhydrous ethanol was slowly added, followed by ultrasonic extraction for 30 min. After centrifugation at 4000 r/min for 10 min, the insoluble substance was washed with 10 mL of 80% ethanol solution and centrifuged. Next, 50 mL of water was added, and the mixture was extracted by ultrasound at 120 W for 30 min. This process was repeated twice. After filtration, the supernatant was transferred to a 200 mL volumetric flask. The residue was washed three times, the washing solution was transferred to a volumetric flask, and then, water was added to the volume, after which 1.0 mL of phenol solution was added to 1 mL of test solution, and 5.0 mL of sulfuric acid was quickly added. After standing for 10 min, the reaction solution was thoroughly mixed and placed in a 30 °C water bath for 20 min. Finally, the absorbance of the reaction solution was measured at 490 nm. The polysaccharide content in the sample was calculated according to Equation (8):

(8) $$\omega =\frac{m_{1}\times V_{1}}{m_{2}\times V_{2}}\times 0.9\times 10^{-4}\times 100\%$$

In the equation, ω is the polysaccharide content in the sample (%), $m_{1}$ is the sugar content (μg) in the sample measurement solution obtained from the standard curve, $V_{1}$ is the constant volume (mL) of the sample, $m_{2}$ is the sample mass (g), $V_{2}$ is the volume (mL) of the sample measurement solution taken during colorimetric measurement, and 0.9 is the correction factor for converting glucose to glucan.

(9) Total flavones

The content of total flavones was measured using the rutin colorimetry method [35]. Samples weighing 500 g were mixed with 50% ethanol in a ratio of 1:35 and extracted by shaking at 60 °C for 3 h. After vacuum filtration, it was concentrated by rotary evaporation at 40 °C to obtain a crude extract of total flavonoids without alcohol. Then, 5 mL of the solution was accurately pipetted into 25 mL volumetric flasks and mixed with 1 mL of 5% NaNO_2_ solution. After 5 min, 1 mL of 10% Al(NO_3_)_3_ solution was accurately added and shaken well. After 6 min, 4 mL l mol/L of NaOH solution was added. Finally, the solution was diluted with 30% ethanol to the mark and heated in a warm water bath for 10 min. After colour development, the absorbance of the solution was measured at 510 nm. The total flavonoid content of the sample was calculated based on the rutin standard curve.

(10) Total polyphenols

The content of total polyphenols was measured using the ferrous tartrate colorimetry method [36]. Samples weighing 5 g were mixed with 40 mL of petroleum ether and then subjected to reflux extraction for 120 min. The solid residue after filtration was mixed with 60% ethanol in a ratio of 1:10 (m/V) at 60 °C and then subjected to ultrasonic extraction for 60 min. The filtered filtrate was diluted to 100 mL, and 20 mL of the solution was mixed with 5.0 g of pre-treated D-101 macroporous resin and placed on an oscillator for 240 min. The adsorption resin after filtration was eluted with 70% ethanol. The eluent was diluted to 50 mL with 70% ethanol. Its total polyphenol content was then determined using ferrous tartrate colorimetry. Gallic acid was used as a standard, and the absorbance at a wavelength of 543 nm was measured. The total polyphenol content in the extract was calculated using a regression equation.

#### 3.2.3. Colour Measurement

The colour changes of HMB under different drying methods were measured by a colour difference meter (NS810, Sannshi Technology Co., Ltd., Shenzhen, China). L value was the lightness index, reflecting the comprehensive value of whiteness and brightness. The values of a* and b* were chromaticity indexes. Each sample was tested eight times in parallel, and three parts (cap, stem, and foot) were measured for each sample. ΔE was the total colour difference value, indicating the colour difference between the dried sample and the control. BI was the browning index. They were calculated by the following Equations (9) and (10):

ΔE = [(L_0_ − L*)^2^ + (a_0_ − a*)^2^ − I − (b_0_ − b*)^2^]^1/2^ (9)

BI = 100(x − 0.31)/0.17 (10)

In the Equation (10),

x = (a* + 1.75 L*)/(5.645 L*+ a* − 3.012 b*) (11)

L_0_, a_0_, and b_0_ refer to the colour parameters of fresh HMB, and L*, a*, and b* refer to that of dried HMB.

#### 3.2.4. Texture Measurement

The hardness and chewiness of the samples were measured with a texture analyser (TA.XT Express, British Stable Micro Systems Corporation, London, UK). The A/mors probe was used, and the trigger force was set as 15 g. The speed before, during, and after measurement was 2 mm/s, 1 mm/s, and 10 mm/s, respectively. For each drying method, 15 samples were taken for repeated assays. For each sample, three parts of the fruiting body (cap, joint, and stem) were measured, and the average value was taken.

#### 3.2.5. Microstructure Analyses

Dried HMB prepared by different drying methods were cut into appropriate sizes with blades. Their cross-sections were palletized and fixed on the sample table. After gold plating twice by an ion sputtering instrument, the samples were placed under a scanning electron microscope (AMARA-KYKY-1000B, Scientific Instrument Factory of Chinese Academy of Sciences, Beijing, China) for microstructure observation.

#### 3.2.6. Grey Relational Analysis

The weights of the influencing factors of 13 indexes, including protein, reducing sugar, fat, crude ash, crude fibre, total flavonoids, total polyphenols, crude polysaccharides, amino acids, ΔE, BI, hardness, and chewiness, were determined by the coefficient of variation method. Then, through data dimensionless, calculation of the absolute difference between the reference series and comparison series, and grey correlation coefficient, the weighted correlation degree was calculated, and the weighted correlation result was used as a reference basis for the final comprehensive evaluation.

### 3.3. Statistical Analysis

The test data were analysed by variance using the statistical analysis software SPSS 22.0. Multiple comparisons were performed by Duncan’s method, and the significance level was set as *p* < 0.05. HMB’s quality characteristics were comprehensively evaluated using the grey correlation analysis method.

## 4. Conclusions

In the present study, the effects of four different drying methods on HMB’s physical and nutritional properties were studied, and the grey correlation analysis method was used to evaluate these methods. H-VFD better retained functional components such as polysaccharides and polyphenols. HPD was conducive to preserving protein and reducing sugar and other nutrients, and HAD maintained a high content of total flavonoids. H-VFD and UH-VFD were beneficial for retaining the original colour, and their products had a looser structure. The results showed that the HMB treated by UH-VFD had the highest-weighted correlation degree, followed by H-VFD. Considering that H-VFD had apparent advantages over UH-VFD in terms of time and energy consumption, H-VFD was selected as a more appropriate method to produce high-quality dried HMB.

## Figures and Tables

**Table 1:** Amino acid composition of HMB prepared by different drying methods (%).

Amino Acid	NH-VFD	H-VFD	HPD	HAD
Essential amino acid (EAA)	7.78 ± 0.04 b	7.89 ± 0.14 b	8.32 ± 0.05 a	8.58 ± 0.16 a
Threonine	0.86 ± 0.02 b	0.88 ± 0.01 b	0.95 ± 0.02 a	0.94 ± 0.01 a
Valine	0.87 ± 0.02 b	0.88 ± 0.01 b	0.96 ± 0.01 a	0.98 ± 0.01 a
Methionine	2.46 ± 0.03 b	2.5 ± 0.04 b	2.46 ± 0.02 b	2.63 ± 0.03 a
Isoleucine	0.69 ± 0.01 b	0.69 ± 0.01 b	0.76 ± 0.02 a	0.79 ± 0.03 a
Leucine	1.13 ± 0.02 b	1.15 ± 0.03 b	1.23 ± 0.02 a	1.27 ± 0.03 a
Phenylalanine	0.8 ± 0.02 b	0.81 ± 0.04 b	0.90 ± 0.03 a	0.93 ± 0.03 a
Lysine	0.97 ± 0.03 c	0.98 ± 0.02 bc	1.06 ± 0.02 a	1.04 ± 0.02 ab
Nonessential amino acids (NEAA)	10.18 ± 0.07 c	10.33 ± 0.06 b	11.75 ± 0.04 a	11.59 ± 0.08 a
Aspartic acid	1.59 ± 0.04 b	1.6 ± 0.02 b	1.58 ± 0.03 b	1.77 ± 0.02 a
Serine	0.89 ± 0.01 b	0.92 ± 0.03 b	1.02 ± 0.03 a	0.99 ± 0.02 a
Glutamate	2.72 ± 0.03 d	2.8 ± 0.03 c	3.42 ± 0.03 a	3.09 ± 0.02 b
Glycine	0.84 ± 0.02 b	0.85 ± 0.04 b	0.94 ± 0.02 a	0.96 ± 0.02 a
Alanine	1.15 ± 0.01 c	1.16 ± 0.02 c	1.62 ± 0.01 a	1.55 ± 0.03 b
Cystine	0.3 ± 0.02 b	0.3 ± 0.01 b	0.34 ± 0.02 a	0.38 ± 0.02 a
Tyrosine	0.49 ± 0.02 a	0.48 ± 0.02 a	0.52 ± 0.03 a	0.53 ± 0.02 a
Histidine	0.33 ± 0.02 a	0.33 ± 0.03 a	0.35 ± 0.03 a	0.35 ± 0.03 a
Arginine	1.16 ± 0.01 a	1.18 ± 0.03 a	1.16 ± 0.04 a	1.12 ± 0.03 a
Proline	0.71 ± 0.02 b	0.71 ± 0.02 b	0.8 ± 0.04 a	0.85 ± 0.02 a
Total amino acids (TAA)	17.96 ± 0.09 b	18.22 ± 0.19 b	20.07 ± 0.04 a	20.17 ± 0.04 a
EAA/TAA	0.43 ± 0.01 a	0.43 ± 0.01 a	0.41 ± 0.01 b	0.43 ± 0.01 a
EAA/NEAA	0.77 ± 0.01 a	0.76 ± 0.01 ab	0.71 ± 0.01 c	0.74 ± 0.01 b

Different letters in the same indicators indicate significant differences (*p* < 0.05).

**Table 2:** Weights of various indicators used in comprehensive evaluation.

Index	Average Value	Standard Deviation	Variation Coefficient	Weighting
Protein	25.13	1.12	0.04	0.019
Reducing sugar	1.90	0.16	0.09	0.037
Fat	3.93	0.60	0.15	0.066
Ash	8.60	0.41	0.05	0.020
Crude fibre	7.43	0.88	0.12	0.051
Total flavonoid	0.42	0.05	0.11	0.046
Total phenolic	0.72	0.05	0.07	0.030
Crude polysaccharide	6.21	0.64	0.10	0.044
Amino acids	19.11	1.18	0.06	0.026
Colour	35.88	21.99	0.61	0.263
Browning index	45.06	32.65	0.72	0.311
Hardness	701.64	92.20	0.13	0.056
Chewiness	965.06	70.03	0.07	0.031

**Table 3:** Results of nondimensionalization.

R_n_ (i)	R_0_ (i)	R_1_ (i)	R_2_ (i)	R_3_ (i)	R_4_ (i)
Protein	1.000	0.912	0.943	1.000	0.996
Reducing sugar	1.000	0.905	0.810	1.000	0.905
Fat	1.000	1.206	1.382	1.029	1.000
Ash	1.000	0.911	0.922	1.000	0.989
Crude fibre	1.000	1.074	1.015	1.000	1.279
Total flavonoid	1.000	0.875	0.813	0.792	1.000
Total phenolic	1.000	0.936	1.000	0.910	0.846
Crude polysaccharide	1.000	0.890	1.000	0.776	0.898
Amino acids	1.000	0.890	0.903	0.995	1.000
Colour	1.000	1.000	1.119	3.489	3.416
Browning index	1.000	1.000	1.072	4.467	4.580
Hardness	1.000	1.000	1.053	1.280	1.294
Chewiness	1.000	1.054	1.164	1.154	1.000

**Table 4:** Absolute value between the reference sequence and comparative sequence.

Δ_n_ (i)	Δ_1_ (i)	Δ_2_(i)	Δ_3_(i)	Δ_4_(i)
Protein	0.088	0.057	0.000	0.004
Reducing sugar	0.095	0.190	0.000	0.095
Fat	0.206	0.382	0.029	0.000
Ash	0.089	0.078	0.000	0.011
Crude fibre	0.074	0.015	0.000	0.279
Total flavonoid	0.125	0.188	0.208	0.000
Total phenolic	0.064	0.000	0.090	0.154
Crude polysaccharide	0.110	0.000	0.224	0.102
Amino acids	0.110	0.097	0.005	0.000
Colour	0.000	0.119	2.489	2.416
Browning index	0.000	0.072	3.467	3.580
Hardness	0.000	0.053	0.280	0.294
Chewiness	0.054	0.164	0.154	0.000

**Table 5:** Grey relational coefficients and grey correlation degrees between reference and comparative sequence.

ξ_n_ (i)	ξ_1_ (i)	ξ_2_(i)	ξ_3_ (i)	ξ_4_ (i)
Protein	0.088	0.057	0.000	0.004
Reducing sugar	0.095	0.190	0.000	0.095
Fat	0.206	0.382	0.029	0.000
Ash	0.089	0.078	0.000	0.011
Crude fibre	0.074	0.015	0.000	0.279
Total flavonoid	0.125	0.188	0.208	0.000
Total phenolic	0.064	0.000	0.090	0.154
Crude polysaccharide	0.110	0.000	0.224	0.102
Amino acids	0.110	0.097	0.005	0.000
Colour	0.000	0.119	2.489	2.416
Browning index	0.000	0.072	3.467	3.580
Hardness	0.000	0.053	0.280	0.294
Chewiness	0.054	0.164	0.154	0.000
Weighted correlation degree	0.978	0.945	0.620	0.620

**Table 6:** Comparison of components between *Hypsizygus marmoreus* by-products and commodity specifications (%).

Components	HMB	HMCS	HMB/HMCS
Protein	26.60	19.10	139.27
Reducing sugar	1.70	1.80	94.44
Fat	4.70	2.80	167.86
Ash	8.30	6.80	122.06
Crude fibre	6.90	6.60	104.55
Total flavonoids	0.39	0.43	90.70
Total phenolics	0.78	0.49	159.18
Crude polysaccharide	6.90	6.27	110.05
Total amino acids	18.22	14.19	128.40

## Data Availability

Data are contained within the article.

## References

[1] Fortune Business Insights (2023). Mushroom Market Size, Share & COVID-19 Impact Analysis, by Type, by Form, and Regional Forecast, 2021–2028. https://www.fortunebusinessinsights.com/industry-reports/mushroom-market-100197.

[2] Antunes F., Taofiq O., Morais A. (2020). Valorization of mushroom by-products as a source of value-added compounds and potential applications. Molecules.

[3] Xu H., Bian C., Wang J. (2010). Determination of nutritional components in three kinds of edible. J. Anhui Agric. Sci..

[4] Li X., Yang Y., Zhou F. (2015). Nutritional contents and flavor substances in fruit bodies and leftovers of *Pleurotus eryngii*. Mod. Food Sci. Technol..

[5] Chang J., Li X., Liang X., Feng T., Sun M., Song S., Yao L., Wang H., Hou F. (2023). Novel umami peptide from *Hypsizygus marmoreus* hydrolysate and molecular docking to the taste receptor T1R1/T1R3. Food Chem..

[6] Chen T., Zhang W., Yuxin Liu Y., Song Y., Wu L., Liu C., Wang T. (2022). Water status and predictive models of moisture content during drying of soybean dregs based on LF-NMR. Molecules.

[7] Gómez-Mejía E., Sacristán I., Rosales-Conrado N., León-González M.E., Madrid Y. (2022). Effect of storage and drying treatments on antioxidant activity and phenolic composition of lemon and clementine peel extracts. Molecules.

[8] Kręcisz M., Stępień B., Pasławska M., Popłoński J., Dulak K. (2021). Physicochemical and quality properties of dried courgette slices: Impact of vacuum impregnation and drying methods. Molecules.

[9] Liu Z., Zielinska M., Yang X., Yu X., Chen C., Wang H., Wang J., Pan Z., Xiao H. (2021). Moisturizing strategy for enhanced convective drying of mushroom slices. Renew. Energy.

[10] Shams R., Singh J., Dash K., Hussain Dar A. (2022). Comparative study of freeze drying and cabinet drying of button mushroom. Appl. Food Res..

[11] Kalinke I., Ulrich Kulozik U. (2023). Irreversible thermochromic ink in the identification of over- and under-processed product segments in microwave-assisted freeze drying. J. Food Eng..

[12] Huang X., Chen X., Wang W., Ge Y., Xie J. (2020). Shelf-life prediction of chilled *Penaeus vannamei* using grey relational analysis and support vector regression. J. Aquat. Food Prod. Technol..

[13] Jiang M. (2021). Sustainable agriculture and food production in Qinghai: Analysis based on grey correlation model. IOP Conf. Ser. Earth Environ. Sci..

[14] Li S., Hu Y., Popov E. (2022). Grey correlation analysis of milling temperature and milling vibration of TC4 titanium alloy. Noise Vib. World..

[15] Urbelis J., Coope J. (2021). Migration of food contact substances into dry foods: A review. Food Addit. Contam. Part A.

[16] Han S., Wang W., Yuan G. (2019). Effect of different drying methods on quality of *Dendrobium officinale* stems. Food Sci..

[17] Deng Y., Tang Q., Zhang R. (2017). Effects of different drying methods on the nutrition and physical properties of *Momordica charantia*. Sci. Agric. Sin..

[18] Besaliev I., Panfilov A., Karavaytsev Y., Reger N., Kholodilina T. (2021). Content of prolin and essential amino acids in spring wheat grain in dry conditions. IOP Conf. Ser. Earth Environ. Sci..

[19] Smith K. (2021). Amino acid overview: Understanding umami. Environ. Nutr..

[20] FAO (1970). Amino acid content of foods and biological data on proteins. Food policy and food Sci. Serv. Nutr. Div..

[21] Zhang L., Wang Z., Yang H. (2016). Effects of different drying methods on the quality of Chinese chestnut. J. Nucl. Agric. Sci..

[22] Liu Y., Zhang Z., Hu L. (2022). High efficient freeze-drying technology in food industry. Crit. Rev. Food Sci..

[23] Krokida M., Maroulis Z. (1997). Effect of drying method on shrinkage and porosity. Dry. Technol..

[24] Saklar S., Ungan S., Katnas S. (2003). Microstructural changes in hazelnuts during roasting. Food Res. Int..

[25] Bao X., Min R., Zhou K., Traffano-Schiffo M.V., Dong Q., Luo Q. (2023). Effects of vacuum drying assisted with condensation on drying characteristics and quality of apple slices. J. Food Eng..

[26] Abdelshafy A.M., Belwal T., Liang Z., Wang L., Li D., Luo Z., Li L. (2022). A comprehensive review on phenolic compounds from edible mushrooms: Occurrence, biological activity, application and future prospective. Crit. Rev. Food Sci..

[27] Lai P., Tang B., Li Y., Wu L., Weng M., Chen J. (2021). Grey correlation analysis for physical and nutritional quality of *Hypsizygus marmoreus* from different drying methods. J. Nucl. Agric. Sci..

[28] (2016). National Health and Family Planning Commission of the People’s Republic of China. National Food Safety Standards, Determination of Protein in Food.

[29] (2016). National Health and Family Planning Commission of the People’s Republic of China. National Food Safety Standards, Determination of Amino Acids in Food.

[30] (2016). National Health and Family Planning Commission of the People’s Republic of China. National Food Safety Standards, Determination of Reducing Sugar in Food.

[31] (2016). National Health and Family Planning Commission of the People’s Republic of China. National Food Safety Standards, Determination of Fat in Food.

[32] (2016). National Health and Family Planning Commission of the People’s Republic of China. National Food Safety Standards, Determination of Ash in Food.

[33] (2003). Ministry of Health of the People’s Republic of China. Determination of Crude Fiber in Plant Food.

[34] (2015). General Administration of Quality Supervision. Inspection and Quarantine of the People’s Republic of China. Determination of Crude Polysaccharides in Plant Derived Food for Export by Phenol Sulfuric Acid Method.

[35] Lai P., Lai F., Chen J. (2015). Studying on purification of total flavonoids from *Perennial lablab* sp. and its bacteriostatic activities. J. Nucl. Agric. Sci..

[36] Ouyang Y., Chen X., Tang H. (2009). Extraction and separation of total polyphenols from *Herba gei*. Food Sci..

